# The analysis about the metastases to Gastrointestinal tract: a literature review, 2000-2023

**DOI:** 10.3389/fonc.2025.1552932

**Published:** 2025-04-17

**Authors:** Ting-Ting Sun, Fu-Guo Liu

**Affiliations:** ^1^ Department of Gastroenterology, The Affiliated Hospital of Qingdao University, Qingdao, Shandong, China; ^2^ Department of Gastroenterology, Qingdao Medical College of Qingdao University, Qingdao, Shandong, China

**Keywords:** gastrointestinal tumors, tumor metastasis, secondary, endoscopy, breast cancer

## Abstract

**Background:**

Cancers of the gastrointestinal tract exhibit a high detection rate, ranking as the fifth most common malignant tumor and the fourth leading cause of cancer-related death. In addition to primary malignant tumors of the gastrointestinal tract, secondary metastatic tumors significantly impact patient survival. The differentiation between primary and secondary gastrointestinal tumors remains a critical issue requiring further research and analysis.

**Methods:**

This is a retrospective, observational study conducted from 2000 to 2023. We systematically searched the literature in PubMed, EMBASE, and COCHRANE databases from January 1, 2000, to November 31, 2023. Patients diagnosed with gastrointestinal (GI) tract metastasis were included in the study.

**Results:**

A total of 165 patients were enrolled in this study. The most prevalent primary tumors were breast cancer (50.30%), renal cancer (16.96%), lung cancer (16.36%), melanoma (12.72%), and liver cancer (3.63%). The median interval between the diagnosis of the primary tumor and the detection of GI metastatic lesions was 8.53 years (range: 1–25 years). The most frequent endoscopic finding was a solitary mucosal or submucosal lesion situated in the gastric body. Metastases to extra-gastrointestinal organs were observed in the majority of patients. The integration of endoscopic biopsy with pathological and immunohistochemical analyses is essential for identifying the tumor origin. Surgical intervention in patients lacking extra-gastrointestinal metastases may improve prognosis.

**Conclusions:**

Breast, renal, lung, liver cancer, and melanoma were identified as the most frequent primary tumors. Clinical symptoms and endoscopic features were unable to predict the primary sites, which still require immunohistochemical analysis for accurate identification. The intervention modality and the presence or absence of distant metastasis significantly influenced patient prognosis.

## Graphical Abstract

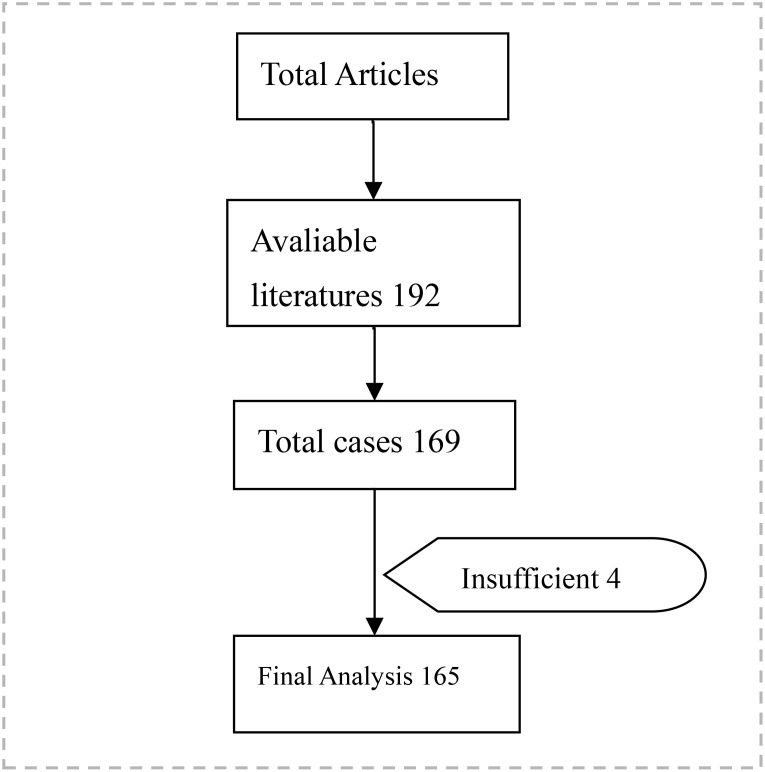

## 1 Introduction

Cancer is a global public health problem and remains one of the leading causes of death globally (1). The global burden of cancer is steadily increasing. According to the latest statistics from the Global Cancer Observatory (https://gco.iarc.fr/), lung cancer (LC) has the highest global incidence among primary cancers at 12.4%, followed by breast cancer (BC) (11.5%), colorectal cancer (CC) (9.6%), prostate cancer (PC) (7.3%), and gastric cancer (GC) (4.8%). In addition to primary tumors, metastatic tumors also pose a significant burden on patient survival (2, 3).

The occurrence of secondary malignant tumors in the gastrointestinal (GI) tract is relatively rare. The stomach is the most common site of metastasis within the GI tract (4). Metastasis to other GI organs, such as the colon, small intestine, or esophagus, occurs at a much lower incidence. According to current data, breast cancer (BC), lung cancer (LC), renal cell cancer (RCC), malignant melanoma (MM), and hepatocellular cancer (HCC) are the most common primary tumors associated with GI tract metastases, with breast cancer being the most prevalent (5, 6).

## 2 Materials and methods

We conducted a literature search in the PubMed, EMBASE, and COCHRANE databases from 1 January 2000 to 31 October 2023, employing an advanced strategy that incorporated the following MeSH terms and keywords: gastrointestinal tumors, tumor metastasis, and gastrointestinal endoscopy. Given that metastases to the gastrointestinal tract are rare, all data were extracted from the literature, including case reports and case series. There were no selection criteria based on age or sex. Published cases were required to document at least one of the following criteria: age, symptoms, the origin of the primary tumor, or survival time. Studies were included only if they contained all the necessary information required to conduct the analysis. Only literature published in the English language was included. The exclusion criteria were defined as literature not published in English. Literature lacking sufficient information was excluded from the analysis.

The selection of articles was based on a review of the study abstracts and full-text articles. Individual articles were excluded if they did not provide sufficient information. Review articles and studies lacking a pooled analysis that describes individual patients were excluded. The data extracted from the literature were archived using Microsoft Excel 2010, and references were managed using Endnote X9.2 reference software. Descriptive and inferential statistical analyses were conducted using SPSS software (version 27; IBM Corp., Armonk, NY, USA). Statistical data were utilized to describe the general characteristics of the patients. Normality was assessed using the Shapiro-Wilk normality test, with p < 0.05 indicating a significant deviation from normality. Differences between groups were analyzed using the Mann-Whitney U test, the χ^2^ test, and Fisher’s exact probability test. P-values less than or equal to the alpha level (α = 0.05) were deemed statistically significant. Two evaluators independently reviewed the titles, abstracts, and full texts of the available literature, screened them against the inclusion and exclusion criteria, and extracted data from the studies. After reviewing and screening, cross-checking was performed. Any disagreements were addressed through discussion or by consulting third-party opinions. The collected data should include the demographic and clinical characteristics of the patients (e.g., age, gender, primary tumor type), the interval between recurrences, the morphological and pathological characteristics of the metastatic tumors, and survival outcomes.

## 3 Results

### 3.1 Basic data

An initial database search identified 222 articles. The included articles comprised case reports and case series. From a total of 192 articles identified, 165 cases with comprehensive data were ultimately included in the analysis. The study population comprised 64 males and 101 females, with a mean age of 62.76 years (range: 30–89 years). The mean age of males was 64.58 years (95% CI: 61.91–67.25), while the mean age of females was 61.59 years (95% CI: 59.20–63.98). The difference in recurrence age between males and females was not statistically significant (p = 0.108).

### 3.2 Primary tumor

The most common primary malignancy resulting in gastrointestinal (GI) metastasis was breast cancer (50.30%), followed by renal cancer (16.96%), lung cancer (16.36%), melanoma (12.72%), and liver cancer (3.63%) (**Table 1**).

**Table 1:** Basical characteristic of patients (n=165).

Characteristic	N	%
Sex		
Female	101	61.21
Male	64	38.78
Age		
<45	13	7.8
45-55	34	21.06
55-65	50	30.30
>65	68	40.21
Primary Tumor		
Breast	83	50.30
Renal	28	16.96
Liver	6	3.63
Melanoma	21	12.72
Lung	27	16.36

Among 77 cases of breast cancer with complete pathological information, invasive ductal adenocarcinoma (IDC) (n=17, 22.07%) and invasive lobular adenocarcinoma. (ILC) (n=60, 77.92%) are the two types of pathology that predominate. In a separate group of 11 lung cancer cases, 10 were identified as non-small cell lung cancer (NSCLC) and only 1 as small cell lung cancer (SCLC). Within the NSCLC cohort, adenocarcinoma accounted for 5 cases (66.7%), squamous cell carcinoma for 3 cases (20%), and large cell lung cancer (LCLC) for 2 cases (13.3%). Among the 21 patients diagnosed with melanoma, 19 presented with melanocytic lesions located in the gastrointestinal tract, while 2 did not have an endoscopic description recorded. We also found that primary melanomas tended to occur on the face, such as the eyes, eyelids, cheeks, or scalp, and to a lesser extent on the feet, vulva, chest, and back. Gastrointestinal metastases from renal cell carcinoma, particularly clear cell carcinoma (ccRCC), are rare (7). While these metastases are typically unilateral(n=20), they can also present bilaterally(n=1). All pathologic types of liver cancer in this study were identified as hepatocellular carcinoma (HCC).

### 3.3 Metastatic interval

The median time interval between the diagnosis of the primary tumor and that of gastrointestinal (GI) metastatic lesions was 8.53 years (range, 1-25 years). In breast cancer patients, this interval was 9.88 years (95% CI, 8.03-11.73 years), while in melanoma patients, it was 7.07 years (95% CI, 3.7-10.30 years). Meanwhile, the median time interval for kidney cancer was 8.09 years (95% CI, 5.58-10.6 years), while for liver tumors, it was 2.25 years (95% CI, 0.72-3.70 years). Lung cancer patients exhibited shorter intervals, predominantly less than 1 year, with specific time points missing for 14 patients (n=14) (**Table 2**). It is evident that the time to secondary metastasis in liver cancer patients is significantly shorter than the average, potentially related to the anatomical location of the liver and its blood supply. The results indicated that the time interval for the occurrence of gastrointestinal metastases significantly differed among tumors of different primary types (p=0.017).

**Table 2:** Media time of metastasis.

Tumor	Time (Years)	95%CI	P-value
Total Breast Melanoma Renal Liver Lung	8.53 9.88 7.07 8.09 2.25 < 1	- 8.03-11.73 3.7-10.30 5.58-10.60 0.72-3.70	0.017

CI, confidence interval.

### 3.4 Gastrointestinal symptoms and endoscopic manifestations

The common endoscopic indications included GI bleeding (54 cases, 23.68%), abdominal pain (51 cases, 22.36%), weight loss (37 cases, 16.22%), nausea and vomiting (30 cases, 13.15%), loss of appetite or early satiety (24 cases, 10.52%), bloating and diarrhea (7 cases, 3.07%), constipation (6 cases, 2.63%), drooling (6 cases, 2.63%), gastrointestinal pain (6 cases), jaundice (3 cases, 1.31%), and no GI symptoms (20 cases, 8.77%) (**Table 3**).

**Table 3:** The symptom of patients.

Symptom	Breast	Liver	Lung	Melanoma	Renal	Total	%
Constipation	5	0	1	0	0	6	2.75
Disphagie	3	0	1	2	0	6	2.75
Abdominal pain	30	1	11	4	4	51	23.39
Weight Loss	25	0	5	4	3	37	16.97
Jaundice	2	0	0	1	0	3	1.38
Nause	26	0	1	3	0	30	13.76
Hemorrhage	13	2	9	9	21	54	24.77
Loss of appetite	17	0	2	2	3	24	11.00
Distention	2	1	2	0	2	7	3.21
Total	123	4	32	32	31	218	

The most common tumor manifestations included primarily elevated submucosal, ulcerative, and diffuse infiltrative lesions (**Table 4**). In cases of BC, endoscopic manifestations include thickening and edema of the mucosa involving the pylorus, leading to gastric retention; a scattered distribution of discoloration-like depressions in the mucosa; and ulceration with distortion or exposure of blood vessels, most commonly found in the lower and middle parts of the gastric body and the gastric horns of the antrum. In the intestines, these manifestations are predominantly located at the distal colon and ileum, typically presenting as multiple scattered, fine, whitish, superficial elevated polypoid lesions with sparse surface vascularity. In cases of HCC, the lesions typically present as larger swellings growing into the cavity, often accompanied by ulcers and ulcerative bleeding, predominantly located in the middle and lower parts of the stomach. In cases of lung cancer (LC), the most common sites are the upper and middle parts of the stomach, the fundus, and the colon, often characterized by prominent masses with crater-like ulcers. Lung metastatic lesions most frequently present as isolated ulcerative lesions in the main body of the stomach, located in the anterior region (8) In cases of melanoma, it predominantly presents as ulcerative foci with melanin deposits of varying sizes, characterized by multiple scattered distributions and a smooth surface mucosa under magnified endoscopy, with a maximum diameter of less than 3 cm. In the intestinal tract, it is primarily found in the terminal ileum, sigmoid colon, and rectum, often appearing as multiple black ulcers with smaller diameters. Gastric metastasis of renal cancer is primarily located in the middle and upper parts of the gastric body, cardia, and gastric fundus, typically characterized by bulging masses with ulceration and blood seepage. In the intestine, it is predominantly observed in the colon, exhibiting similar features.

**Table 4:** Characteristics of the metastatic lesions (n=165).

Characteristic	N	%
Organ		
Colorectal	11	8.39
Jejunum lleum	5	3.81
Duodenum	9	6.87
Stomach	106	80.91
Aspect		
Elevated	71	50.53
Necrosis	2	1.41
Ulcerated	33	23.40
Infiltrative	12	8.51
Edema	4	2.83
Thickening of the folds	5	3.54
Depression	7	4.96
Erosion	4	2.83
Flat	3	2.12

### 3.5 Metastasis to GI tract and other organs

Among the 37 patients with mono-digestive metastasis, 26 (70.27%) cases presented with gastric metastasis only, 8 (21.62%) cases had intestinal metastasis only, and 3 (8.10%) cases exhibited concurrent gastrointestinal metastasis.

Metastases to other organs were observed in 104 (74.3%) cases of the total patient cohort. The most frequently involved organs included bone, followed by the lung, liver, brain, and peritoneum (**Table 5**). Among these patients, isolated intestinal metastases were identified in 17 (16.3%) cases, while gastrointestinal metastases were observed in 8 (7.69%) cases. The remaining 79 (75.96%) cases exhibited metastases confined solely to the stomach. Gastric metastases were observed in various regions, ranging from the fundus to the antrum, including the lesser curvature and posterior wall of the stomach (9).

**Table 5:** The characteristics of extra-GI metastatic.

Sites	Breast	Liver	Lung	Melanoma	Renal	Total	%
Bone	34	0	6	1	2	43	24.85
Appendix	3	0	0	0	0	3	1.73
Peritoneum	12	0	0	0	1	13	7.51
Skin	2	0	2	2	0	6	3.46
Liver	9	0	7	8	2	26	15.02
Eye	2	0	0	0	0	2	1.15
Lung	8	3	0	8	6	25	14.45
Brain	6	1	9	5	3	24	13.87
Diaphragm	2	0	1	0	0	3	1.73
Omentum	6	0	0	0	0	6	4.61
Kindey	3	0	3	3	0	9	5.20
Adrenal Gland	1	0	0	3	1	5	2.89
Thyroid	3	0	0	0	0	3	1.73
Pleura	0	0	4	1	0	5	2.89
Total	91	4	32	31	15	173	100

Regardless of the presence or absence of extra-gastrointestinal metastases, the majority (70%) of metastatic carcinomas were predominantly localized to the stomach (**Table 6**). Patients with lung and kidney cancers demonstrated a relatively high probability of intestinal-only metastases, while melanoma patients were more likely to exhibit both types of gastrointestinal metastases, in contrast to breast cancer patients who presented with gastric-only metastases. However, Darina Kohoutova (10) confirmed that MM exhibits a particular affinity for metastasizing to the small intestine, particularly the jejunum and ileum.

**Table 6:** The metastatic sites in Gastrointestinal Tract.

	Gastric metastases	%	Intestinal metastases	%	Both	%	Total
	N		N		N		N
Breast	45	80.36	7	12.50	4	7.14	56
Liver	3	75.00	1	25.00	0	0.00	4
Lung	13	68.42	4	21.05	2	10.52	19
Melanoma	13	81.25	1	6.25	2	12.50	16
Renal	5	55.55	4	44.40	0	0.00	9
Total	79		17		8		104

### 3.6 Pathological diagnosis

In the vast majority of cases, an endoscopic biopsy combined with pathological and immunohistochemical methods serves as an extremely important adjunct in determining the tumor’s origin. In terms of immunohistochemistry(IHC) of breast, ER expression (n=54, 88.52%), PR expression (n=27, 60%), CK7 expression (n=28, 96.55%), gross cystic disease fluid protein 15 (GCDFP-15) expression (n=28, 96.55%) showed high positivity rate, while human epidermal growth factor receptor 2(Her2) (n=4, 14.81%) had a lower,. In contrast, the IHC expression in the HCC included glypican-3, AFP, CKIMN, and CK8. In lung cancer, the expression levels of TTF-1 (n=9, 81.82%), CK7(n=8, 88.89%), CKAE1(n=5, 100%), and CKAE3(n=5, 100%) were relatively high. Positive expression of CK20, CDX2, and CD34 has not been observed. In MM, there was a very high positivity rate for S100, HMB45 and Mela A, which holds differential significance for determining the primary type of gastrointestinal metastasis. The positive expression of CAM5.2, c-KIT, and Ki67 also played a supplementary role in the differential diagnosis. In renal cancer, CD10 and VI were expressed at higher levels, while CK7 and CK20 were expressed at lower levels.

### 3.7 Recurrence and prognosis

For primary tumors, the mean time to recurrence for patients undergoing surgical treatment after diagnosis was significantly different from that of patients treated conservatively, suggesting that surgical interventions positively impact patients’ prognosis (**Table 7**).

**Table 7:** Operation and recurrence time.

Primary Tumor	Surgery or not	Number of cases	Time (Years)	P-value
Breast	0	10	3.53	0.002
	1	39	10.6	
Liver	0	2	2	—
	1	2	1.3	
Lung	0	4	0.72	—
	1	8	0.70	
Renal	0	0	—	—
	1	23	7.47	
Melanoma	0	1	1	—
	1	19	5.1	

For metastatic tumors, the proportions of patients undergoing surgical intervention and those managed with conservative treatment were comparable. Among the surgical group, 22 patients (73.3%, N=30) survived for more than 1 year postoperatively, compared to 15 patients (48.3%, N=31) in the conservative treatment group who survived beyond 1 year. The 1-year survival rate was significantly higher in the surgical group compared to the conservative treatment group (p=0.046). In breast cancer patients, the 1-year survival rate was significantly higher in the surgical group (94.1%) than in the non-surgical group (66.7%) (p=0.047), suggesting that surgical intervention may improve patient prognosis.

To investigate the impact of extra-gastrointestinal metastasis on survival, we observed that 48.07% of the 52 patients with distant metastases had a 1-year survival probability, compared to 92.30% of the 13 patients without metastases to other sites (p=0.004). These findings indicate that patients with distant metastases exhibit a significantly lower 1-year survival rate compared to those without distant metastases.

## 4 Discussion

According to the latest statistics from CANCER TODAY, lung cancer has the highest incidence rate in China at 22.0%. Arnold M (11) indicates that the incidence of gastrointestinal cancers remains high in certain areas, with an increased risk observed among the younger generation (12). For gastrointestinal metastases, it is crucial to differentiate between primary and secondary tumors, as this distinction significantly impacts patient prognosis and treatment options (13).

According to our statistics, the primary tumors most likely to metastasize to the gastrointestinal tract include breast, kidney, lung, melanoma, and liver tumors. Previous studies have demonstrated that the highest metastatic rates were associated with melanoma, lung, and breast cancers (5). Among primary breast cancers, invasive lobular carcinoma (ILC) is the most common type (14), which consistent with previously discovered articles (15).The majority of lung cancers are classified as NSCLC (16), whereas most liver cancers are categorized as HCC. The primary malignant melanoma predominantly affects the eyes (17) and the skin of the hands and feet (18). All primary RCCs were clear-cell cancers (19).

There is great heterogeneity of gastrointestinal manifestations (20), the majority of patients experienced abdominal discomfort, gastrointestinal bleeding symptoms including hematemesis and melena, weight loss, nausea, and vomiting, with only 8.4% of patients remaining asymptomatic (5). Most patients with breast cancer experience mild symptoms, including nausea, vomiting, and upper abdominal discomfort. When concentrating on breast cancer, there is typically no distinction made between primary and secondary forms (14).Regarding patients with lung cancer, diarrhea, abdominal distension, epigastric discomfort, and malaise are the predominant symptoms (16).RCC is characterized by gastrointestinal bleeding, including hematemesis and melena, as its primary symptoms (5). This suggests that the likelihood of gastrointestinal bleeding in renal cancer patients is elevated. However, there are also metastatic melanomas that present mainly with gastrointestinal bleeding (21).

Most patients admitted to the hospital underwent endoscopy, where the most common endoscopic findings included elevated ulcerative or isolated lesions in the gastric body, Kim, G.H., et al. (22) study indicated that isolated lesions are more prominent than other presentations. Breast cancer metastasis primarily involves gastric metastasis (9). Gastrointestinal metastases secondary to lung cancer predominantly occur in male smokers aged 45 to 90 years. It is hypothesized that gastrointestinal metastases are caused by swallowing cancer-rich sputum that reaches the digestive tract (23, 24). The gastrointestinal manifestations of melanoma metastases with no significant heterogeneity from primary melanoma (25). Reggiani, H.C (18) have shown that secondary metastases of melanoma occur more often in the body of the stomach and to a lesser extent in the gastric sinus. In addition, MM tends to spread to the small intestine (51-71%), especially the jejunum and ileum (26, 27).

Pathological diagnosis continues to be an essential tool for determining the origin of a tumor (28). Our findings indicate that most reported metastatic tumors were poorly differentiated and included even the most malignant undifferentiated signet-ring cell carcinomas (14, 29). For breast cancer, our study highlights that markers extending beyond ER, PR, and HER2—including GATA3, Ki-67, E-cadherin, GCDFP-15, cytokeratin (CK), and mammaglobin—are associated with breast epithelial origin and are commonly utilized as key components of diagnostic panels (14, 30). Notably, GATA3 demonstrates superior sensitivity in detecting distant metastases when compared with GCDFP-15 and mammaglobin, providing enhanced capability in distinguishing primary versus metastatic lesions (31). The characteristic immunophenotypic profile of CK7+/CK20- combined with GATA3+/CDX2- or GATA3+/SATB2- patterns provides definitive evidence supporting metastatic breast carcinoma to gastrointestinal sites (32). Early studies have demonstrated that tumors of pulmonary origin typically display a CK7+/CK20- immunophenotype, while tumors of gastrointestinal origin generally present a distinct CK7-/CK20+ phenotype (8, 33). Current studies indicate that CK7 demonstrates high sensitivity for neoplasms of gastrointestinal or pulmonary origin, with positive staining for CK14 and CK18 being indicative of squamous cell carcinoma and adenocarcinoma, respectively. Furthermore, TTF-1 expression suggests a potential origin from thyroid or lung tumors (33). Subsequently, we adopted a diagnostic approach incorporating CK7, CK14, CK18, CK20, and TTF-1 to aid in tumor origin determination (16). In cases of RCC, studies have demonstrated that RCC is frequently positive for vimentin, keratin, EMA, CD10, Pax2, CAM5.2, and CAIX, while being negative for renal-specific calreticulin and microalbumin (34). In this article, the high expression levels of CD10, vimentin (VI), and CAM5.2 further support the diagnosis of metastatic tumors. Regarding MM, Altay, A.Y (35)shows that HMB45 and Melan-A demonstrate lower sensitivity but higher specificity, whereas S-100 exhibits better sensitivity but lower specificity. In cases of HCC, new biomarkers have yet to be implemented in clinical practice, with alpha-fetoprotein (AFP) remaining the most widely utilized and frequently expressed marker discussed in this study. Consequently, it remains a challenge to develop additional early-specific diagnostic markers (36). IHC not only guides treatment but is also instrumental in differentiating between primary and secondary gastrointestinal tumors (13, 37).

Our statistics indicate that some patients diagnosed with gastrointestinal metastases also have concomitant brain, bone, peritoneal, and adrenal metastases, as confirmed in previous literature (13, 37).The subsequent treatment plan following diagnosis is guided by pathology, immunophenotyping, and symptoms. In breast cancer, markers such as ER, CK7, GCDFP can improve the diagnosis of the disease and guide treatment (38). Patients receiving only systemic supportive therapy or those who are resistant to hormonal or biological agents are likely to derive less benefit from treatment (39).In patients with gastrointestinal metastases originating from lung cancer, the high expression levels of PD-L1 may represent a viable target for immune checkpoint inhibitor therapy (40). Additionally, surgical resection may be a beneficial option for patients experiencing symptoms such as intestinal obstruction, while also highlighting the importance of supportive care (8, 33). Current treatments for renal cancer include surgical intervention, chemotherapy, endocrine therapy, arterial embolization, and palliative care. Molecular targeted therapy has shown a positive effect on advanced or metastatic renal cancer (19). For melanoma patients, there are various treatment modalities such as endoscopy, surgery, chemotherapy and systemic targeted therapy (10). A total of 40-50% of melanomas harbor site-specific mutations that could benefit from targeted therapy (41).

International reports on metastatic cancer of the gastrointestinal tract are limited and often overlooked, despite the consistently high morbidity and mortality associated with gastrointestinal cancers. The detection, diagnosis, and treatment strategies must be tailored to the specific type of tumor identified, necessitating heightened vigilance for patients with a prior history of cancer and more meticulous performance of endoscopies (14). Multi-site biopsies, along with corresponding pathological analyses and IHC, can significantly impact patient outcomes. Additionally, examining other parts of the body, particularly the bones and brain, is crucial for enhancing diagnostic yield.

## 5 Shortcomings

Reflecting on the article, the first problem is that the sample size is insufficient, and due to the limitations of the retrospective study design, part of the information, like follow-up data, is missing or missed, which leads to the limitations of some of the conclusions of the article and the inadequacy of the findings. Secondly, malignant have a very different distribution and manifestation in different regions and ethnicities, so subgroup analyses based on region and ethnicity is an extremely important part of the study. However, the data used in our study were derived from published literature, which is inherently subject to selection bias and lacks comprehensive clinical and pathological data, limiting our ability to conduct high-quality analyses stratified by region or ethnicity. Therefore, more comprehensive and detailed follow-up and data extraction, as well as adequate sample sizes, are necessary in subsequent studies.

## 6 Conclusion

Gastrointestinal metastatic carcinoma is relatively rare, and its metastatic mechanism remains poorly understood. In addition, gastrointestinal metastases exhibit significant heterogeneity in clinical symptoms and endoscopic manifestations, complicating the establishment of standardized diagnostic criteria. Gastrointestinal metastasis is generally regarded as an indicator of advanced disease. Current treatment strategies primarily involve surgery combined with radiotherapy or immunotherapy, while targeted therapy may also be applicable in certain cases. Prognosis varies significantly depending on the primary tumor and metastatic characteristics, highlighting the importance of early diagnosis, accurate identification, and timely treatment of gastrointestinal metastasis. In conclusion, the diagnosis and treatment of gastrointestinal metastatic cancer remain challenging. Future research should focus on elucidating the underlying metastatic mechanisms and developing more effective therapeutic strategies.
